# Supplementary material for: Genomic Divergence of Sympatric Lineages Within Stichopus cf. horrens (Echinodermata: Stichopodidae): Insights on Reproductive Isolation Inferred From SNP Markers
Source: Ecol Evol. 2025 Apr 27;15(4):e71283. doi: 10.1002/ece3.71283 (PMC12034849; doi:10.1002/ece3.71283)
Supplement: Supplementary file 1 — Figures S1–S4. [file ECE3-15-e71283-s002.pdf]

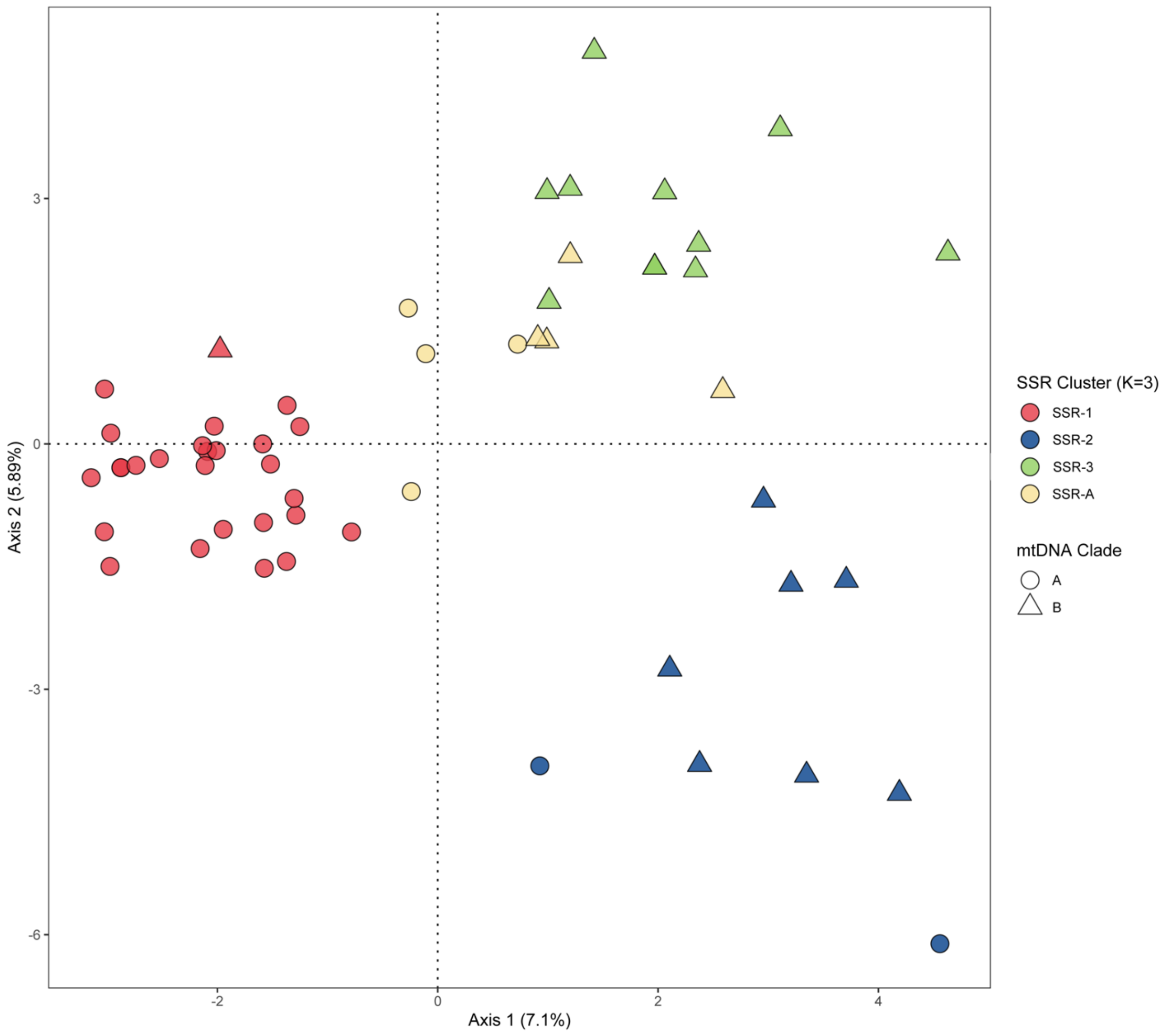

Figure A1. Principal component analysis plot of microsatellite genotypes from 55 *Stichopus* cf. *horrens*. Each point represents an individual, with lineages identified by shape (mtDNA Clade) and color (microsatellite genotype clusters or SSRs). SSR clusters were inferred from STRUCTURE analysis at $K = 3$ groups, with individuals assigned to a cluster when the ancestry coefficient (q) is > 0.9, and admixed if $0.1 < q < 0.9$.

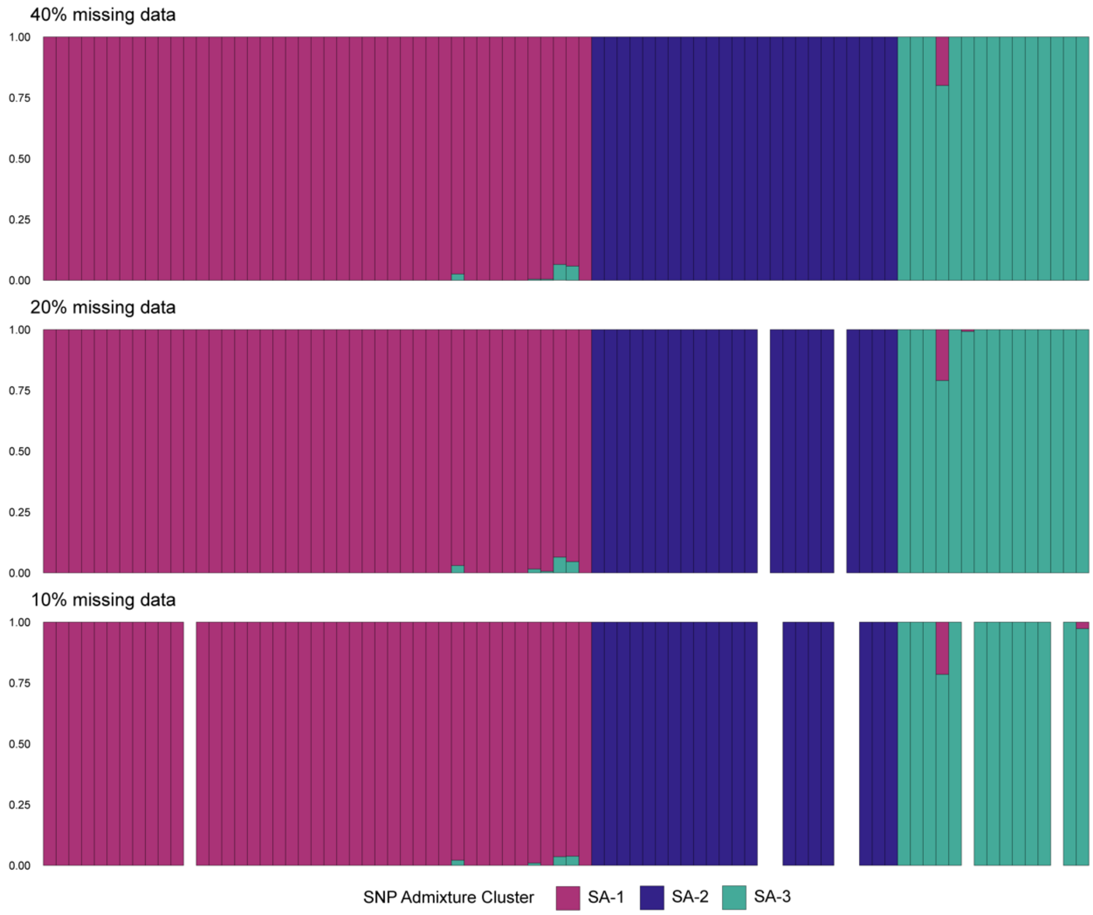

Figure A2. Bar plots of individual ancestry based on Admixture analysis at $K = 3$ genetic clusters across three SNP (Single Nucleotide Polymorphism) datasets with varying levels of missing data (40%, 20%, 10% missing data). Each bar represents one individual, with bar color representing the proportion of ancestry (q) in each genetic cluster. White bars represent individuals which were excluded after filtering for missing data.

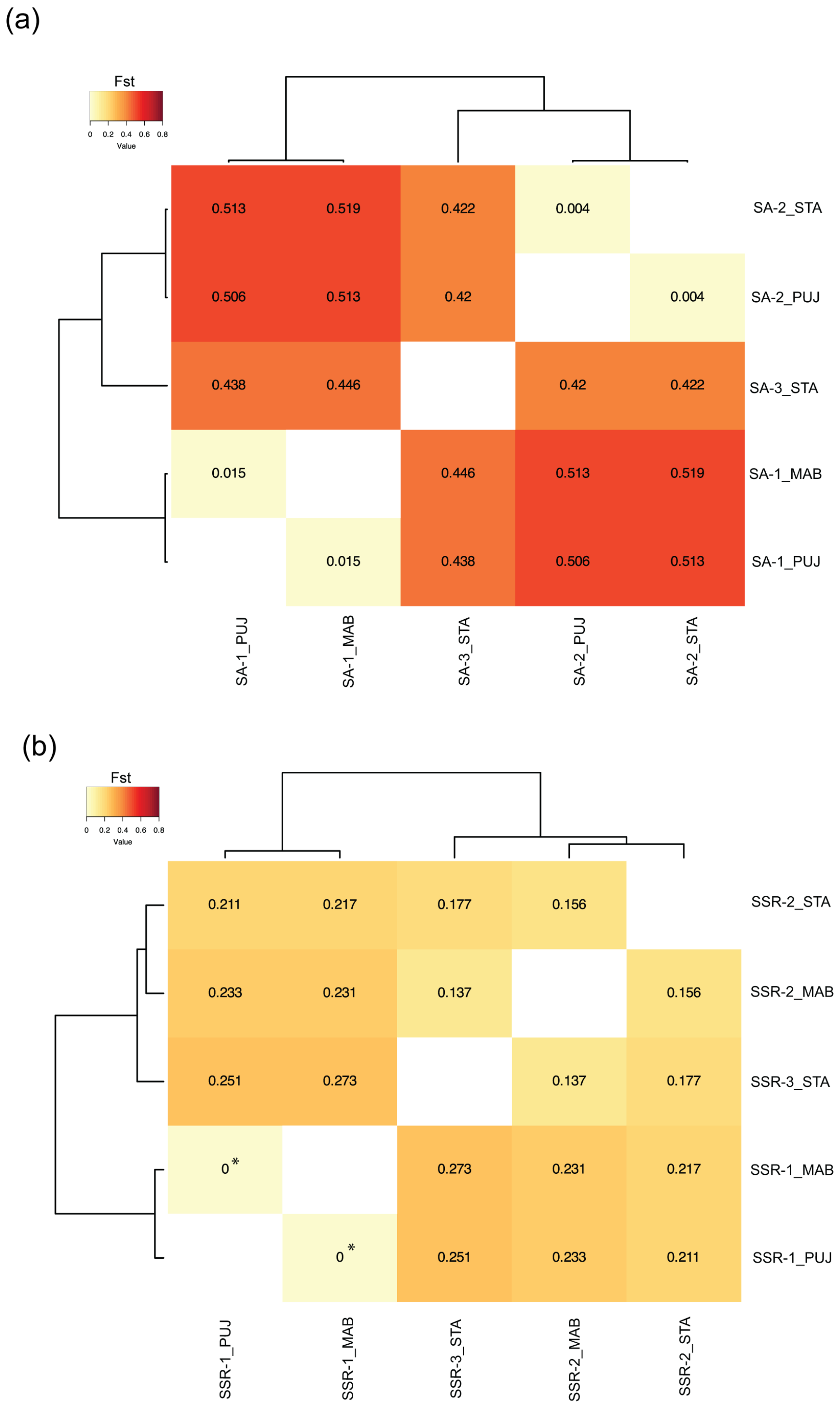

Figure A3. Heatmap of pairwise F_{ST} values for *Stichopus* cf. *horrens* lineages at three collection sites. (a) SNP lineages for 78 individuals based on 9,788 SNP loci. (b) SSR lineages for 44 individuals based on 6 microsatellite loci. Groups with less than five colonies were excluded from the analysis. Hierarchical clustering is represented by the dendrogram, showing three genetic lineages. Non-significant F_{ST} values ($F_{ST} = 0$) are marked with an asterisk.

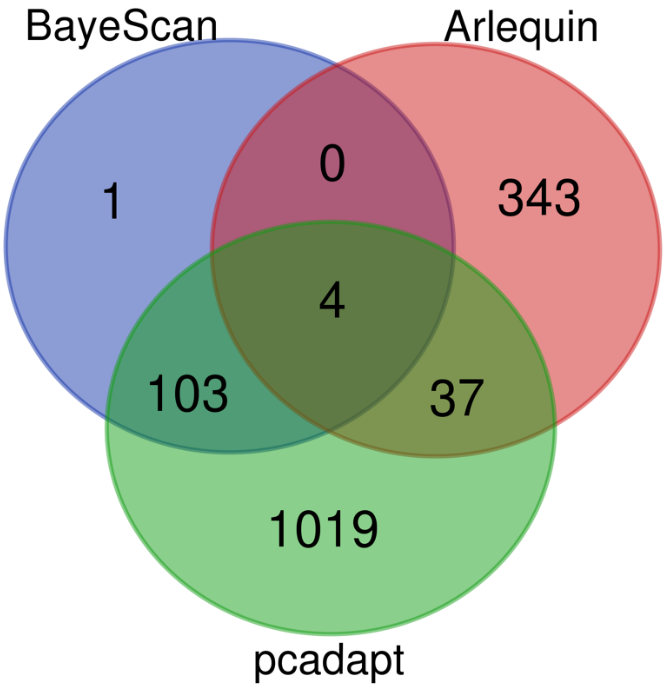

Figure A4. Venn diagram showing the number of outlier SNPs identified across different outlier detection methods and the overlap between these methods.
